# Viral-bacterial coinfection affects the presentation and alters the prognosis of severe community-acquired pneumonia

**DOI:** 10.1186/s13054-016-1517-9

**Published:** 2016-10-25

**Authors:** Guillaume Voiriot, Benoit Visseaux, Johana Cohen, Liem Binh Luong Nguyen, Mathilde Neuville, Caroline Morbieu, Charles Burdet, Aguila Radjou, François-Xavier Lescure, Roland Smonig, Laurence Armand-Lefèvre, Bruno Mourvillier, Yazdan Yazdanpanah, Jean-Francois Soubirou, Stephane Ruckly, Nadhira Houhou-Fidouh, Jean-François Timsit

**Affiliations:** 1 Service de Réanimation Médicale et Infectieuse, Hôpital Bichat Claude Bernard, Hôpitaux Universitaires Paris Nord Val de Seine, Assistance Publique - Hôpitaux de Paris (AP-HP), Paris, France; 2 Service de Virologie, Hôpital Bichat Claude Bernard, Hôpitaux Universitaires Paris Nord Val de Seine, Assistance Publique - Hôpitaux de Paris (AP-HP), Paris, France; 3 Service de Maladies Infectieuses et Tropicales, Hôpital Bichat Claude Bernard, Hôpitaux Universitaires Paris Nord Val de Seine, Assistance Publique - Hôpitaux de Paris (AP-HP), Paris, France; 4 Service de Microbiologie, Hôpital Bichat Claude Bernard, Hôpital Bichat Claude Bernard, Hôpitaux Universitaires Paris Nord Val de Seine, Assistance Publique - Hôpitaux de Paris (AP-HP), Paris, France; 5 Université Paris Diderot-Paris VII, Paris, France; 6 Université de Grenoble 1, Center U823 Epidemioloy of Cancers and Severe Diseases, La Tronche, France; 7 Hôpital Bichat Claude Bernard, 46 rue Henri Huchard, Paris, 75018 France

**Keywords:** Pneumonia, Viral pneumonia, Respiratory viruses, Intensive care

## Abstract

**Background:**

Multiplex polymerase chain reaction (mPCR) enables recovery of viruses from airways of patients with community-acquired pneumonia (CAP), although their clinical impact remains uncertain.

**Methods:**

Among consecutive adult patients who had undergone a mPCR within 72 hours following their admission to one intensive care unit (ICU), we retrospectively included those with a final diagnosis of CAP. Four etiology groups were clustered: bacterial, viral, mixed (viral-bacterial) and no etiology. A composite criterion of complicated course (hospital death or mechanical ventilation > 7 days) was used. A subgroup analysis compared patients with bacterial and viral-bacterial CAP matched on the bacterial pathogens.

**Results:**

Among 174 patients (132 men [76 %], age 63 [53–75] years, SAPSII 38 [27;55], median PSI score 106 [78;130]), bacterial, viral, mixed and no etiology groups gathered 46 (26 %), 53 (31 %), 45 (26 %) and 30 (17 %) patients, respectively. Virus-infected patients displayed a high creatine kinase serum level, a low platelet count, and a trend toward more frequent alveolar-interstitial infiltrates. A complicated course was more frequent in the mixed group (31/45, 69 %), as compared to bacterial (18/46, 39 %), viral (15/53, 28 %) and no etiology (12/30, 40 %) groups (*p* < 0.01). In multivariate analysis, the mixed (viral-bacterial) infection was independently associated with complicated course (reference: bacterial pneumonia; OR, 3.58; CI 95 %, 1.16–11; *p* = 0.03). The subgroup analysis of bacteria-matched patients confirmed these findings.

**Conclusions:**

Viral-bacterial coinfection during severe CAP in adults is associated with an impaired presentation and a complicated course.

**Electronic supplementary material:**

The online version of this article (doi:10.1186/s13054-016-1517-9) contains supplementary material, which is available to authorized users.

## Background

Community-acquired pneumonia (CAP) is a common disease that may become severe, leading to admission to intensive care units (ICU) [1]. CAP etiology is usually bacterial; however, the causative role of respiratory viruses emerged recently [2]. Multiplex polymerase chain reaction (mPCR) kits screen a large panel of respiratory viruses, and nowadays are available in clinical practice. They were used within several studies among adult ICU patients with CAP [3–6]. High rates of positivity were reported, up to 49 % [4], with strong variations in the distribution of viral species according to the population, the season, and the geographic area. However, the causative role of respiratory viruses identified in the respiratory tract during pneumonia is still debatable, since respiratory viruses might be present in asymptomatic adult subjects [7, 8]. Some experimental data focusing on virus-bacteria interactions during respiratory tract infections supported a pathogenic role of respiratory viruses during pneumonia [9]. In mice, the coinfection of influenza with *S. pneumoniae* [10], *L. pneumophila* [11] or *S. aureus* [12] impaired the anti-influenza immune response and increased the mortality. Similar synergistic results are obtained with *S. pneumoniae* and respiratory syncytial virus [13], or *S. pneumoniae* and rhinovirus [14].

In humans, the pathogenic role of respiratory viruses in virus-bacteria coinfected patients remains unclear. We conducted a comprehensive observational study among adult ICU patients with CAP, to compare clinical characteristics, biological presentation, and outcome according to the presence of virus in the respiratory tract.

## Methods

### Study design and patient selection

We conducted a retrospective monocenter observational study in the 26-bed ICU of the Bichat Claude Bernard University Hospital (Paris, France). During the study period, all consecutive patients having undergone a mPCR in the respiratory tract within the 72 hours following their ICU admission were screened. Medical records were independently reviewed by two physicians. All patients with a final diagnosis of pneumonia were included (see definitions for population selection in Additional file 1).

### Data collection

AT ICU admission and during ICU stay, data regarding demographics, comorbidity, clinical examinations, laboratory and radiological findings, microbiologic investigations, and therapeutic management were collected (for details, see Additional file 1). Mortality was defined as death from any cause within 30 days of hospitalization.

Pneumonia severity was assessed through the Pneumonia Severity Index (PSI) [15], and the Simplified Acute Physiologic Score (SAPS) II [16].

### Microbiological evaluation

Respiratory tract specimens underwent Gram staining and quantitative culture for bacterial pathogens. Urine antigen testing of *S. pneumoniae* and *L. pneumophila* used the BinaxNOW kits (Alere, Jouy en Josas, France). The immunoglobulin (Ig) antibodies testing for *C. pneumoniae* and *M. pneumoniae* was considered positive if IgM antibodies were identified or if a significant increase in IgG antibodies was observed between paired serum samples.

The respiratory mPCR were performed either in nasopharyngeal (NP) swabs or in lower respiratory tract (LRT) specimens, usually bronchoalveolar lavage fluid otherwise endotracheal aspirate. During the study period, different mPCR kits were used (for details, see Additional file 1). Respifinder® 19 (Pathofinder, Maastricht, The Netherlands) and Filmarray Respiratory Panel (BioFire Diagnostics, Salt Lake City, UT, USA) could not detect bocavirus nor differentiate rhinovirus and enterovirus; therefore, rhinovirus and enterovirus results were grouped as picornavirus (rhinovirus).

Either in blood samples or in bronchoalveolar lavage fluid, the cytomegalovirus PCR used the CMV R-gene® kit (Argene, Verniolle, France ) or the QS-RGQ® kit (Qiagen, Hilden, Germany), and the herpex simplex virus PCR used LightCycler® HSV (Roche, Basel, Switzerland).

### Classification of patients according to pathogens

A bacterium was considered as a causative pathogen of the pneumonia if this bacterium fulfilled at least one criterion (for details, see Additional file 1). A virus identified with PCR was always considered as a causative pathogen of the pneumonia.

Pneumonia was defined as: (i) bacterial, if microbiological investigations revealed at least one bacterium and no virus; (ii), viral, if microbiological investigations revealed at least one virus and no bacterium; (iii) mixed (virus-bacteria), if microbiological investigations revealed at least one virus and one bacterium; and (iv) no etiology, if microbiological investigations revealed no virus and no bacterium.

### Endpoints

The primary endpoint was to identify presentation and prognosis-specific features in virus-bacteria coinfected patients. Comparisons focused on microbiological data, biological findings and radiological patterns on admission, ICU course and hospital outcome. A composite criterion named “complicated course” included hospital death or mechanical ventilation for more than 7 days. The second endpoint was to describe the epidemiology of respiratory viruses in adult patients admitted to the ICU for a CAP. Patients were clustered into four groups according to the microbiological etiology of pneumonia: bacterial, viral, mixed, and no etiology.

### Matching procedure

To better control for impact of the bacterial pathogen on our main findings, we also designed a subgroup analysis comparing patients with bacterial and mixed viral-bacterial CAP, matched on the bacterial pathogen. If more than one bacterium was identified in cases, we sought for a control with the same bacterial combination.

### Data presentation and statistical analysis

Continuous data were expressed as median [first through third quartiles] and were compared using the Kruskall-Wallis test followed by pairwise Mann-Whitney test. Categorical data were expressed as number (percentages) and were evaluated using the chi-square test or Fisher’s exact test. *p* values less than 0.05 were considered significant. A univariate logistic regression with clinically relevant variables was used to identify variables associated with a complicated course. A multivariate conditional logistic regression, including variables with *p* value less than 0.10 in the previous step, was used to identify variables independently associated with complicated course. Similar statistical analyses were performed to identify variables independently associated with hospital death and mechanical ventilation for more than 7 days in survivors at day 28. Quantitative variables that did not validate the log-linearity assumption were transformed into categorical variable according to their median value. Missing data were imputed to the median or to the more frequent value. The accuracy of the final model was tested using area under the receiver operating characteristic curve analysis and the Hosmer-Lemeshow chi-square test. An additional multivariate conditional logistic regression, limited to bacterial and mixed groups, was performed to search specifically for an association between virus-bacteria coinfection and complicated course. Comparisons in the subgroup analysis of bacteria-matched patients involved univariate conditional logistic regression followed by multivariate conditional logistic regression to assess associations between microbiological diagnosis and complicated course, adjusting for clinically relevant variables. Analyses were performed using the SAS software package (SAS Institute, Cary, NC, USA).

## Results

### Population

**Fig. 1:**
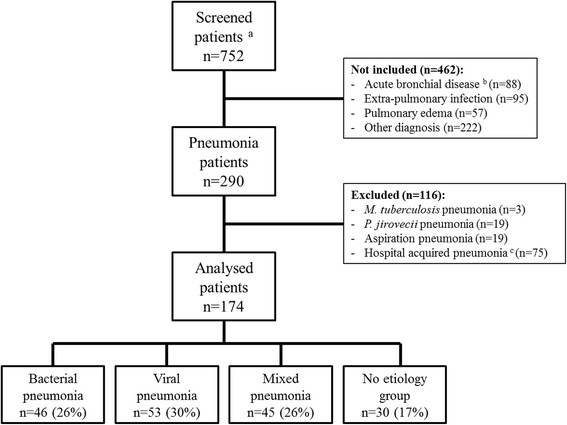
Flow chart. **a** All consecutive patients admitted to the ICU during a 3.5-year period and having undergone a mPCR on a respiratory tract sample within 72 hours following ICU admission were screened. **b** Acute bronchial disease included COPD exacerbation, asthma, and acute bronchitis. **c** Pneumonia was considered hospital-acquired if neither clinically present nor in an incubation period at time of hospital admission

From October 2011 to June 2015, 752 patients were screened (Fig. 1). The final study group consisted of 174 patients (132 men (76 %), age 63 [53–75] years, SAPS II 38 [27;55]) (Table 1). One third (33.3 %) were referred from medical wards or another ICU. The median PSI score at hospital admission was 106 [78;130]. Pneumonia was considered health care-associated pneumonia in nearly half the patients (49.4 %). At least one factor of immunosuppression was present in 32.8 % of patients.

**Table 1:** Baseline characteristics, behavior during ICU stay, and outcome of 174 patients with severe CAP, according to the microbiological diagnosis

Patients	All patients (n = 174)	Bacterial group (n = 46)	Viral group (n = 53)	Mixed group (n = 45)	No etiology group (n = 30)	*p* value^a^
Age, y	63 [53;75]	64 [53;75]	64 [54;75]	63 [54;75]	66 [57;78]	0.85
Sex, male	132 (75.9)	37 (80.4)	38 (71.7)	33 (73.3)	24 (80)	0.69
Weight, kg	71 [62;82]	70 [60;80]	71 [64;84]	70 [60;77]	75 [62;83]	0.64
Smoking	53 (31.7)	16 (37.2)	16 (30.8)	13 (30.2)	8 (27.6)	0.83
McCabe score > 1	38 (21.8)	7 (15.2)	13 (24.5)	12 (26.7)	6 (20)	0.56
WHO performance status > 0	53 (33.5)	10 (22.7)	19 (41.3)	14 (35.9)	10 (34.5)	0.30
Chronic immunosuppression	57 (32.8)	16 (34.8)	21 (39.6)	14 (31.1)	6 (20)	0.32
HIV	14 (8)	7 (15.2)	3 (5.7)	3 (6.7)	1 (3.3)	0.20
Steroid therapy	19 (10.9)	2 (4.3)	10 (18.9)	6 (13.3)	1 (3.3)	0.06
Other immunosuppressive	21 (12.4)	3 (6.5)	10 (20)	7 (15.6)	1 (3.4)	0.08
Solid organ transplantation	14 (8)	2 (4.3)	5 (9.4)	6 (13.3)	1 (3.3)	0.31
Cancer	15 (8.6)	6 (13)	4 (7.5)	2 (4.4)	3 (10)	0.51
Chronic disease^b^	86 (49.4)	20 (43.5)	34 (64.2)	20 (44.4)	12 (40)	0.08
Coronary artery disease	27 (15.5)	6 (13)	9 (17)	9 (20)	3 (10)	0.64
HCAP^c^	86 (49.4)	22 (47.8)	31 (58.5)	22 (48.9)	11 (36.7)	0.29
Transfer from another ward^d^	58 (33.3)	14 (30.4)	19 (35.8)	17 (37.8)	8 (26.7)	0.72
Antibiotics before ICU admission^e^	77 (44.3)	15 (32.6)	30 (56.6)	19 (42.2)	13 (43.3)	0.12
Organ failures on ICU admission						
Glasgow < 15	42 (24.1)	14 (30.4)	11 (20.8)	11 (24.4)	6 (20)	0.66
Shock	32 (18.4)	14 (30.4)	3 (5.7)	11 (24.4)	4 (13.3)	<0.01
PaO_2_/FIO_2_ ratio	174 [130;230]	173 [130;229]	172 [122;227]	165 [134;228]	200 [165;252]	0.22
SAPS II score	38 [27;55]	39 [32;60]	36 [26;48]	46 [34;59]	33 [18;46]	0.02
PSI score at hospital referral	106 [78;130]	110 [84;152]	98 [82;128]	119 [98;126]	89 [70;121]	0.12
PSI class IV-V at hospital referral	114 (65.5)	31 (67.4)	33 (62.3)	36 (80)	14 (46.7)	0.03
Organ supports during ICU stay						
Noninvasive ventilation	55 (31.8)	14 (30.4)	21 (40.4)	12 (26.7)	8 (26.7)	0.44
Mechanical ventilation	98 (56.3)	28 (60.9)	22 (41.5)	36 (80)	12 (40)	<0.01
ARDS	60 (34.5)	17 (37)	13 (24.5)	22 (48.9)	8 (26.7)	0.06
Dialysis	37 (21.3)	10 (21.7)	10 (18.9)	12 (26.7)	5 (16.7)	0.72
Vasopressors	80 (46.2)	22 (47.8)	19 (36.5)	27 (60)	12 (40)	0.12
Outcome						
Length of mechanical ventilation, d	9 [5;13]	6.5 [3;12.5]	7 [4;12]	9 [6;14]	10 [7.5;17.5]	0.34
Follow-up duration, d^f^	15 [10 ; 29]	14 [5;23]	18 [12;32]	16 [11;31]	14.5 [12;19]	0.25
Hospital mortality	30 (17.2)	6 (13)	6 (11.3)	13 (28.9)	5 (16.7)	0.10
Complicated course^g^	74 (42.5)	18 (39.1)	15 (28.3)	31 (68.9)	10 (33.3)	<0.01

Data are presented as median [first through third quartiles] or number (%)

*ARDS* acute respiratory distress syndrome, *CAP* community-acquired pneumonia; HCAP health care-associated pneumonia, *HIV* human immunodeficiency virus, *ICU* intensive care unit, *PSI* Pneumonia Severity Index, *SAPS* Simplified Acute Physiologic Score, *WHO* World Health Organization.

^a^ *p* values refer to differences between bacterial, viral, mixed, and no etiology groups in univariate logistic regression

^b^Chronic disease included chronic dialysis, mellitus diabetes requiring oral medication and/or insulin, chronic heart failure classified NYHA 3 or 4, cirrhosis, chronic respiratory failure requiring long-term oxygen therapy, and chronic immunosuppression

^c^Pneumonia was considered health care-associated (HCAP) in the presence of at least one of the following conditions: hospitalization for ≥ 2 days in the preceding 90 days, institutionalization, chronic dialysis, and chronic immunosuppression

^d^Transfer from another ward included transfers from another ICU and from the medical wards

^e^Antibiotics before ICU admission referred to any administration of antibiotics, whatever drug regimen, before the ICU referral, i.e., in emergency departments or in other medical wards

^f^The follow-up duration was defined as the time between the date of the mPCR and the date of hospital discharge. If the patient was deceased in hospital, the date of death was considered hospital discharge. If the patient was not deceased in hospital and the date of hospital discharge was not available, the date of ICU discharge was considered hospital discharge ^g^Complicated course was defined as hospital death and/or mechanical ventilation > 7 days

### Microbiological diagnosis

The microbiological investigations are displayed in Additional file 1: Table S1. The microbiological findings are displayed in Table 2. mPCR was performed in NP swabs exclusively (n = 110, 63.2 %) or in LRT specimen exclusively (n = 43, 24.7 %) or both (n = 21, n = 13.2 %). Respiratory tract specimens for bacterial culture have been obtained in 153 (87.9 %) patients. In 34 (19.5 %) patients the only respiratory tract specimen that has been obtained was sputum. Near half the patients (n = 77, 44.3 %) received antibiotics prior to referral.

**Table 2:** Microbiological findings of 174 patients with severe CAP

Patients	All patients (n = 174)	Bacterial group (n = 46)	Viral group (n = 53)	Mixed group (n = 45)	No etiology group (n = 30)
*S. pneumoniae*	40 (23)	19 (41.3)	-	21 (46.7)	-
Other streptococci	6 (3.4)	2 (4.3)	-	4 (8.9)	-
*S. aureus*	12 (6.9)	6 (13)	-	6 (13.3)	-
*L. pneumophila*	8 (4.6)	7 (15.2)	-	1 (2.2)	-
*C. pneumoniae – M. pneumoniae*	6 (3.4)	5 (10.9)	-	1 (2.2)	-
*H. influenzae*	13 (7.5)	5 (10.9)	-	8 (17.8)	-
*Enterobacteriaceae* species	11 (6.3)	7 (15.2)	-	4 (8.9)	-
*P. aeruginosa*	7 (4)	2 (4.3)	-	5 (11.1)	-
Other bacteria	3 (1.7)	1 (2.2)	-	2 (4.4)	-
Mixed flora	10 (5.7)	6 (13)	-	4 (8.9)	-
Picornavirus (rhinovirus)^a^	22 (12.6)	-	7 (13.2)	15 (33.3)	-
Influenza A	32 (18.4)	-	19 (35.8)	13 (28.9)	-
Influenza B	6 (3.4)	-	4 (7.5)	2 (4.4)	-
Parainfluenza	3 (1.7)	-	2 (3.8)	1 (2.2)	-
Respiratory syncytial virus	9 (5.2)	-	5 (9.4)	4 (8.9)	-
Human metapneumovirus	12 (6.9)	-	6 (11.3)	6 (13.3)	-
Coronavirus	14 (8)	-	7 (13.2)	7 (15.6)	-
Adenovirus	3 (1.7)	-	2 (3.8)	1 (2.2)	-
Bocavirus	1 (0.6)	-	1 (1.9)	0 (0)	-
Cytomegalovirus	1 (0.6)	-	1 (1.9)	0 (0)	-
Herpes simplex virus	3 (1.7)	-	1 (1.9)	2 (4.4)	-
Varicella zoster virus	1 (0.6)	-	1 (1.9)	0 (0)	-

Data are presented as number (%)

*CAP* community-acquired pneumonia

^a^Picornavirus included rhinovirus and enterovirus

A microbiological documentation was obtained in 144 (82.8 %) patients. At least one bacterium was identified in 91 (52.3 %) patients and at least one virus in 98 (56.3 %) patients. Bacterial documentation was obtained in 56 (57.7 %) patients who had not been exposed to antibiotics prior to referral, compared to 35 (45.5 %) antibiotics-exposed patients (*p* = 0.13). *S. pneumoniae* was the most commonly identified bacterium, found in 40 (23 %) patients. Of these 40 patients, *S. pneumoniae* was cultured in blood in five patients. In 22 (12.6 %) patients, more than one bacterial species was identified. Taken together, *Pseudomonas aeruginosa* and *Enterobacteriaceae* species were identified in nine (19.5 %) patients of the bacterial group and nine (19 %) patients of the mixed group.

Influenza viruses and picornavirus (rhinovirus) were the most commonly identified viruses, found in 38 (21.8 %) and 22 (12.6 %) patients, respectively. In nine (5.2 %) patients, more than one virus was identified. In the 21 patients having undergone mPCR in both NP swabs and LRT specimen, the mPCR were discordant in ten patients, including eight patients with a NP mPCR positive and a LRT mPCR negative and two patients with a NP mPCR negative and a LRT mPCR positive.

### Analysis according the microbiological diagnosis

The four study groups did not differ in terms of demographics, comorbid conditions, chronic immunosuppression, HCAP factors, incidence of transfer from another ward, and antibiotics before referral (Table 1). The microbiological investigations also were similar (Additional file 1: Table S1). *S. pneumoniae* was the predominant bacterium in both bacterial and mixed groups (Table 2). Only one patient was infected with extended-spectrum beta-lactamase-producing *Enterobacteriaceae*. Fourteen patients were infected with intracellular bacteria, including *L. pneumophila*, *C. pneumoniae* and *M. pneumoniae*, but only two had a viral coinfection. Influenza viruses were the most commonly viruses in the viral group, whereas picornavirus (rhinovirus) and influenza viruses were equally predominant in the mixed group.

The incidence of shock was higher in patients with a documented bacterial infection (30.4 % and 24.4 % in bacterial and mixed group, respectively) in comparison with other patients (5.7 % and 13.3 % in viral and no etiology groups, respectively, *p* < 0.01) (Table 1). Overall, patients with mixed pneumonia displayed a higher disease severity on hospital and ICU admission, with higher rate of PSI class IV-V at hospital referral (80 % vs. 67.4 %, 62.3 % and 46.7 % in bacterial, viral, and no etiology groups, respectively, *p* = 0.03) and increased SAPS II (46 vs 39, 36 and 33 in bacterial, viral, and no etiology groups, respectively, *p* = 0.02).

Among the 45 patients with mixed pneumonia, 30 were infected with viruses other than influenza, including 14 patients infected with picornavirus (rhinovirus).

Virus-infected patients displayed high serum levels of creatine kinase, a low platelet count and a trend toward a high incidence of alveolar-interstitial infiltrates on chest X-ray (Additional file 1: Table S2). No significant difference was observed between groups regarding cardiac troponin T. However, 20 (20.4 %) virus-infected patients displayed a cardiac troponin T above the upper limit of normal, compared to eight (10.5 %) virus-uninfected patients.

All the bacteria-infected patients, except one in the bacterial group (45/46, 97.8 %) and one in the mixed group (44/45, 97.8 %), received an appropriate antimicrobial regimen within the first 24 hours of ICU admission. Among the influenza-infected patients, most patients (32/38, 84.2 %) received ozeltamivir within the first 24 hours of admission to ICU. Complicated course was more frequent in the mixed group (31/45, 68.9 %), as compared to bacterial (18/46, 39.1 %), viral (15/53, 28.3 %), and no etiology (12/30, 40 %) groups (*p* < 0.01). In multivariate analysis, the microbiological diagnosis was identified as an independent factor of complicated course (Table 3). The microbiological diagnosis was not independently associated with hospital death (Additional file 1: Table S3) but with mechanical ventilation for more than 7 days in survivors at day 28 (Additional file 1: Table S4). The additional multivariate analysis, limited to bacterial and mixed groups, identified the virus-bacteria coinfection as independently associated with the complicated course (reference: bacterial pneumonia; OR, 3.56; CI 95 %, 1.24–10.18; *p* = 0.02) (Table 4). The impact of the viral coinfection was similar for influenza and other viruses (Additional file 1: Table S5). The subgroup analysis of bacteria-matched patients confirmed these findings (Additional file 1: Table S6).

**Table 3:** Multivariate analysis of the risk factors for complicated course in 174 patients with severe CAP

Variables	OR	95 % CI	*p* value
Microbiological diagnosis			
Bacterial pneumonia	Ref	…	
Viral pneumonia	0.69	0.24–1.95	0.48
Mixed pneumonia	3.15	1.12–8.83	0.03
No etiology pneumonia	1.29	0.40–4.21	0.67
Coronary artery disease	3.52	1.22–10.15	0.02
Shock on ICU admission	4.63	1.56–13.74	0.006
Lactate dehydrogenase > 245 U/L	4.27	1.55–11.78	0.005
PSI class IV-V at hospital referral	4.67	1.96–11.12	0.0005

*CAP* community-acquired pneumonia, *ICU* intensive care unit, *OR* odds ratio, *PSI* Pneumonia Severity Index, *Ref* reference, *95 % CI* = 95 % confidence interval

**Table 4:** Risk factors for complicated course in patients with severe CAP: multivariate analysis exploring the 91 patients with either bacterial or mixed viral-bacterial infection

Variables	OR	95 % CI	*p* value
Microbiological diagnosis			
Bacterial pneumonia	Ref	…	
Mixed pneumonia	3.56	1.24–10.18	0.02
Coronary artery disease	2.59	0.60–11.19	0.20
Shock on ICU admission	5.63	1.53–20.73	0.009
Lactate dehydrogenase > 245 U/L	5.16	1.49–17.90	0.01
PSI class IV-V at hospital referral	2.69	0.76–9.51	0.13

*CAP* community-acquired pneumonia, *ICU* intensive care unit, *Ref* reference, *OR* odds ratio, *PSI* Pneumonia Severity Index, 95 % CI 95 % confidence interval

## Discussion

This retrospective study investigated the impact of the mixed viral-bacterial coinfection on the presentation and outcome of ICU patients with CAP. Real-time mPCR tests identified at least one virus in the respiratory tract of 56.3 % of patients. Specific biological and radiographic features, including high serum levels of creatine kinase, a low platelet count, and a high incidence of alveolar-interstitial infiltrates were observed in these patients, who presented also the higher status severity on hospital admission and the higher frequency of hemodynamic and respiratory failures during ICU stay. The viral-bacterial coinfection was independently associated with a complicated course. These findings were confirmed by a subgroup analysis comparing bacteria-infected and virus-bacteria coinfected patients.

In this study, more than one patient out of two (56.3 %) were infected with at least one virus, in line with a recent report on ICU ventilated patients with CAP [4]. This finding illustrated the high yield of an aggressive diagnostic strategy with a broad panel mPCR on respiratory tract specimens. Elsewhere, the rate of viral documentation reported in adult ICU patients with CAP was slightly lesser, from 23 to 49 % [3–5].

Respiratory tract specimens for bacterial test were recovered in a high proportion of patients (87.9 %), including a LRT specimen in 68.4 % of patients. Bacterial documentation was obtained in 52.3 % of patients, in the range of other studies on ICU patients with CAP (36–82 %) [3, 4]. Interestingly, the rate of bacteremia, 6.3 %, was markedly lower than usually observed [17, 18]; it might be attributable to a high pre-referral exposure to antibiotics (44.3 %). Overall, 82.8 % of patients had a microbiological diagnosis, a high rate in line with that of Karhu and colleagues [4].

The predominant viruses were influenza viruses and picornavirus (rhinovirus) (21.8 % and 12.6 %, respectively). Previous studies in ICU patients with CAP reported a much lower rate of influenza infection, from 2 to 10 % [3–6]. It might be explained by low influenza vaccine coverage in our population. Unfortunately, the influenza vaccination history was usually not available in medical records, preventing any conclusion on this point. The incidence of rhinovirus was consistent with that of previous reports [3, 5]. Interestingly, a “viral phenotype” is emerging, mainly characterized by high serum levels of creatine kinase, and high frequency of alveolar-interstitial infiltrates on chest X-ray. It was not yet reported, since previous works having largely used mPCR in ICU patients with CAP did not specifically record biology and radiographic data [3–6]. Chest X-ray patterns, notably ground glass opacities, were consistent with previous data [19]. Elevated levels of serum creatine kinase could be attributable to the rhabdomyolysis associated with viral infections, especially with influenza and parainfluenza viruses [20]. The trend toward a high serum level of cardiac troponin T in virus-infected patients might suggest acute myocardial injury. Furthermore, alveolar-interstitial infiltrates could indicate pulmonary edema. Unfortunately, brain natriuretic peptide dosage and echocardiogram data were not available, although left ventricular dysfunction has been previously described in influenza A infection [21].

Virus-bacteria coinfection was observed in one patient out of four, consistently with previous reports (9–39 %) [3, 4, 22]. It was identified as independently associated with a complicated course. This finding is original, since previous works that studied CAP patients requiring ICU admission did not point out any relationship between viral-bacterial coinfection and severity [3, 4]. Karhu and colleagues studied a limited cohort (n = 49), whereas Choi and colleagues observed a low rate of virus-bacteria coinfected patients in their cohort (18/198, 9 %), thus preventing any analysis on outcome in both studies. In hospitalized patients with CAP, some data suggested that the viral-bacterial coinfection might be associated with high-risk classes of PSI [23], higher length of hospital stay [24], and higher mortality [25]. More specifically, *S. pneumoniae-*influenza and *S. pneumoniae*-rhinovirus coinfections were correlated with a more severe illness in hospitalized patients [22, 26, 27]. In our study, in order to avoid overinterpreting the data, we decided to consider respiratory viruses as a homogeneous group of pathogens. This might be criticized, since the pathogenicity probably differs from one viral species to another. However, our results remained similar when taking into account non-influenza viruses only. Further studies with larger populations may explore this point, with comparing the prognosis of CAP patients according to the type of virus as well as the different virus-bacteria combinations. Our findings might have a therapeutic impact. Currently, only very few medications targeting non-influenza respiratory viruses are available in clinical practice (cidofocir, ribavirine, immunoglobulins). But some novel antiviral drugs, targeting mainly respiratory syncytial virus and parainfluenza virus, have shown promising results in immunocompromised patients [28, 29] and in human volunteers [30]. Whether these upcoming drugs would be of interest in virus-infected CAP patients requiring ICU admission is questionable. We identified the virus-bacteria coinfected patients to be at risk of complicated ICU course, so further studies might explore potential benefits of the upcoming antiviral drugs in this high-risk population.

Our study has several limitations. First, this is a monocenter study, so the generalization of our results should be cautious. Second, this study included patients with pneumonia that required ICU admission. It means that we did not study mild to moderate pneumonia, preventing any conclusion on this population. Third, the study was retrospective so we did not control the microbiological investigations. By definition, a mPCR was performed in the respiratory tract of every included patient because it was the criteria for patient screening. But some other microbiological testings were only occasionally performed, i.e. cytomegalovirus and herpes simplex virus PCR, or *C. pneumoniae* and *M. pneumoniae* antibodies testing. Furthermore, the retrospective design prevented us obtaining a number of data, which were rarely reported in medical records by physicians, including vaccine history, symptoms before hospital referral, and duration of symptoms before ICU admission. Fourth, only patients having undergone a mPCR in the respiratory tract within the 72 hours following their ICU admission were screened; this might suggest a confounding of indication. Fifth, we chose a composite endpoint, decided a priori. Indeed, considering the predictable low hospital mortality, we did not choose hospital death as primary endpoint, because the low frequency of the event (death) would have favored the absence of significant difference between groups. Sixth, we made the assumption that a virus identified with PCR was a causative pathogen of the pneumonia. This might be criticized since respiratory viruses might be present in asymptomatic adult subjects [7, 8]. Seventh, almost half the patients (n = 77, 44.3 %) received antibiotics prior to ICU admission. Thus some patients may have had false-negative findings regarding bacterial infection and may have been falsely included in the viral group instead of the mixed group or to the no etiology group instead of the bacterial group.

## Conclusions

Viruses are frequently identified in the respiratory tract of patients with pneumonia requiring ICU admission, with a strong predominance of influenza and rhinovirus. The viral-bacterial coinfection concerns more than a quarter of patients and is associated with an impaired radiological and biological presentation and with a complicated course.

## Additional file

Additional file 1: Table S1. Microbiological investigations performed in 174 patients with severe CAP. **Table S2.** Initial biological findings and radiological patterns of 174 patients with severe CAP, according to the microbiological diagnosis. **Table S3.** Multivariate analysis of the risk factors for hospital death in 174 patients with severe CAP. **Table S4.** Multivariate analysis of the risk factors for mechanical ventilation for more than 7 days in survivors at day 28. **Table S5.** Baseline characteristics, behavior during ICU stay, and outcome of 45 patients with mixed infection, according to the viral diagnosis. **Table S6.** Baseline characteristics, initial biological findings and radiological patterns, ICU course and outcome in bacteria-matched patients with severe CAP. (DOCX 39 kb)

## Abbreviations

ARDS: acute respiratory distress syndrome
AST: aspartate aminotransferase
CAP: community-acquired pneumonia
CI: confidence interval
COPD: chronic obstructive pulmonary disease
HCAP: health care-associated pneumonia
HIV: human immunodeficiency virus
ICU: intensive care unit
Ig: immunoglobulin
LRT: lower respiratory tract
mPCR: multiple polymerase chain reaction
NP: nasopharyngeal
OR: odds ratio
PSI: Pneumonia Severity Index
Ref: reference
SAPS II: Simplified Acute Physiology Score
WHO: World Health Organization
