# Supplementary material for: Viral-bacterial coinfection affects the presentation and alters the prognosis of severe community-acquired pneumonia
Source: Crit Care. 2016 Oct 25;20:375. doi: 10.1186/s13054-016-1517-9 (PMC5112669; doi:10.1186/s13054-016-1517-9)
Supplement: Additional file 1: Table S1. — Microbiological investigations performed in 174 patients with severe CAP. Table S2. Initial biological findings and radiological patterns of 174 patients with severe CAP, according to the microbiological diagnosis. Table S3. Multivariate analysis of the risk factors for hospital death in 174 patients with severe CAP. Table S4. Multivariate analysis of the risk factors for mechanical ventilation for more than 7 days in survivors at day 28. Table S5. Baseline characteristics, behavior during ICU stay, and outcome of 45 patients with mixed infection, according to the viral diagnosis. Table S6. Baseline characteristics, initial biological findings and radiological patterns, ICU course and outcome in bacteria-matched patients with severe CAP. (DOCX 39 kb) [file 13054_2016_1517_MOESM1_ESM.docx]

**Additional file 1**

**Material and Methods**

**Definitions used for patient selection:**

Pneumonia was defined as the association of i) at least two signs of systemic inflammation response; ii) a new infiltrate evidenced in the chest radiograph; and iii) a recent onset of at least two of the following signs of pneumonia: cough, sputum production, chest pain, crackles, respiratory distress, hypoxemia (oxygen saturation below 90% in ambient air or requirement of oxygen therapy up to 5 liters per minute or mechanical ventilation), and fever. We excluded patients with *Mycobacterium tuberculosis* infection, *Pneumocystis jirovecii* pneumonia and aspiration pneumonia, and patients whom pneumonia was hospital-acquired, that meant neither clinically present nor in incubation period at time of hospital admission.

**Data collection**

Data regarding demographics, comorbidity, McCabe score and World Health Organization Performance status, initial clinical, laboratory and radiological findings, microbiological investigations during the ICU stay, therapeutic management before the ICU referral and during the ICU stay, ICU and hospital lengths of stay, and ICU and hospital discharge status (dead or alive), were collected. Septic shock upon admission was collected. A transfer from another ward was defined as transfer from another ICU or from the medical wards. Antibiotics before ICU admission referred to any administration of antibiotics before the ICU referral. We collected the need for mechanical ventilation and its duration, the need for renal replacement therapy and for vasopressors.

Underlying chronic disease was defined by at least one of the following comorbid conditions: chronic dialysis, mellitus diabetes, NYHA 3 or 4 chronic heart failure, cirrhosis, chronic respiratory failure, and chronic immunosuppression (defined by at least one of the following conditions: splenectomy, chronic neutropenia (<0.5 G/L), HIV infection, long term steroid therapy, other long term immunosuppressive therapy, solid organ transplantation, and malignant hemopathy or tumor).

The duration between the date of the mPCR and the date of hospital discharge defined the follow-up period. Mortality was defined as death from any cause within 30 days of hospitalization. If the patient was not deceased in hospital and the date of hospital discharge was not available, the date of ICU discharge was considered for hospital discharge. In case of death at hospital, the date of death was considered for hospital discharge.

Pneumonia was considered health-care associated (HCAP) in the presence of at least one of the following conditions: hospitalization for ≥ 2 days in the previous 90 days, institutionalization, chronic dialysis, and chronic immunosuppression.

**Microbiological evaluation using mPCR**

During the study period, different mPCR kits were used: the Respifinder® 19 (Pathofinder®, Maastricht, Netherlands) from May 2011 to February 2012, Respifinder® 22 (Pathofinder®, Maastricht, Netherlands) from March 2012 to December 2013 and AnyplexTM II RV16 (Seegene®, Seoul, South Korea) from January to June 2015. The Filmarray Respiratory Panel (BioFire Diagnostics®, Salt Lake City, USA) was also used upon specific request. Of note, the Respifinder® 22 and the Filmarray Respiratory panel included respectively four bacterial targets (*L. pneumophila*, *M. pneumoniae*, *C. pneumoniae* and *B. pertussis*) and three bacterial targets (*M. pneumoniae*, *C. pneumoniae* and *B. pertussis*).

**Definition of bacterial causative pathogen for pneumonia**

A bacterium was considered as causative pathogen of the pneumonia if this bacterium fulfilled at least one criteria among the followings: i) *S. pneumoniae* or *S. aureus* or *L. pneumophila*, whatever type of testing and level of positivity; ii) identified in pleural fluid or blood; iii) *Chlamydiae pneumoniae* or *Mycoplasma pneumoniae* identified with Ig antibodies testing; iv) *Bordetella pertussis* or *C. pneumoniae* or *M. pneumoniae* identified by mPCR; v) identified within a sputum specimen with good-quality criteria (leucocyte > 25/field and epithelial cells < 10/field) and ≥ 10^6^ colony-forming units/mL); vi) identified within bronchoalveolar lavage fluid at ≥ 10^4^ colony-forming units/mL or protected distal sample at ≥ 10^3^ colony-forming units/mL, or bronchial aspirate specimen at ≥ 10^5^ colony-forming units/mL. When more than two bacteria were identified, the term “mixed flora” was used.

**Table S1.** Microbiological investigations performed in 174 patients with severe CAP.

| Patients | All  patients  (n=174) | Bacterial  group  (n=46) | Viral  group  (n=53) | Mixed  group  (n=45) | No etiology  group  (n=30) | P  Value ^a^ |
| --- | --- | --- | --- | --- | --- | --- |
| mPCR in nasopharyngeal swabs ^b^ | 131 (75.3) | 34 (73.9) | 38 (71.7) | 32 (71.1) | 27 (90) | 0.23 |
| mPCR in LRT specimen ^b^ | 64 (37.2) | 16 (35.6) | 20 (38.5) | 21 (46.7) | 7 (23.3) | 0.23 |
| Bronchoalveolar lavage | 53 (30.8) | 15 (33.3) | 16 (30.2) | 16 (35.6) | 6 (20) | 0.53 |
| Endotracheal aspirate | 11 (6.4) | 1 (2.2) | 4 (7.7) | 5 (11.1) | 1 (3.3) | 0.35 |
| Bacterial culture of respiratory sample |  |  |  |  |  |  |
| Sputum | 61 (35.1) | 15 (32.6) | 20 (37.7) | 11 (24.4) | 15 (50) | 0.14 |
| Distal protected sample | 58 (33.5) | 15 (32.6) | 13 (24.5) | 21 (47.7) | 9 (30) | 0.11 |
| Bronchial aspirate | 27 (15.7) | 8 (17.4) | 8 (15.4) | 7 (15.9) | 4 (13.3) | 0.97 |
| Bronchoalveolar lavage | 78 (44.8) | 22 (47.8) | 22 (41.5) | 24 (53.3) | 10 (33.3) | 0.34 |
| *S. pneumoniae* urine antigen testing | 151 (86.8) | 39 (84.8) | 46 (86.8) | 39 (86.7) | 27 (90) | 0.93 |
| *L. pneumophila* urine antigen testing | 149 (85.6) | 38 (82.6) | 45 (84.9) | 39 (86.7) | 27 (90) | 0.83 |
| Pleural fluid culture | 9 (5.2) | 4 (9.1) | 1 (1.9) | 1 (2.2) | 3 (10) | 0.19 |
| Blood culture | 172 (98.9) | 45 (97.8) | 52 (98.1) | 45 (100) | 30 (100) | 0.67 |

Data are presented as number (%). CAP= Community-Acquired Pneumonia; LRT = Lower Respiratory Tract; mPCR = multiplex Polymerase Chain Reaction.

^a^ P values refer to differences between bacterial, viral, mixed and no etiology groups in univariate logistic regression.

^b^ mPCR was performed in nasopharyngeal swabs exclusively (n=110, 63.2%), in LRT specimen exclusively (n=43, 24.7%), or in both specimen (n=21, n=13.2%).

**Table S2.** Initial biological findings and radiological patterns of 174 patients with severe CAP, according to the microbiological diagnosis.

| Patients | All  patients  (n=174) | Bacterial  group  (n=46) | Viral  group  (n=53) | Mixed  group  (n=45) | No etiology  group  (n=30) | P  value ^a^ |
| --- | --- | --- | --- | --- | --- | --- |
| Initial biological findings |  |  |  |  |  |  |
| Neutrophils, G/L | 10.1 [6.4;13.8] | 11 [7.3;17.4] | 9.3 [5.6;12.9] | 8.6 [3.8;13.7] | 12.3 [9;16.8] | 0.07 |
| Lymphocytes, G/L | 0.9 [0.5;1.4] | 0.8 [0.5;1.4] | 0.9 [0.6;1.3] | 0.7 [0.5;1.4] | 1.1 [0.5;1.3] | 0.37 |
| Platelets, G/L | 206 [138;272] | 212.5 [180;266] | 171 [116;223] | 202 [129;273] | 261 [203;315] | <0.01 |
| Procalcitonin, µg/L | 2 [0.4;18.5] | 4.2 [0.6;36.5] | 1.2 [0.4;3.3] | 4.9 [0.6;47.8] | 0.9 [0.3;8.6] | <0.01 |
| Creatine kinase, U/L | 188 [87;486] | 127 [74;338] | 231 [96;460] | 381 [143;829] | 154.5 [45;259] | 0.02 |
| Lactate dehydrogenase, U/L | 396 [255;586] | 356 [202;508] | 395 [264;593] | 489.5 [333;638] | 340 [215;502] | 0.11 |
| AST, U/L | 39 [26;71] | 42 [27;60] | 37 [27;70] | 47 [32;100] | 27 [21;62] | 0.05 |
| Bilirubin total, µmol/L | 12 [7;18] | 13 [8;19] | 11 [7;17] | 13 [8;18] | 10 [7;15] | 0.18 |
| Cardiac troponin T, > 0.35 ng/mL | 28 (19.7) | 3 (7.7) | 13 (31) | 7 (18.4) | 5 (21.7) | 0.07 |
| Initial radiologic patterns |  |  |  |  |  |  |
| Bilateral infiltrates | 112 (64.4) | 30 (65.2) | 34 (64.2) | 27 (60) | 21 (70) | 0.85 |
| Alveolar-interstitial infiltrates | 84 (50) | 15 (33.3) | 32 (61.5) | 23 (54.8) | 14 (48.3) | 0.06 |
| Pleural effusion | 9 (5.2) | 3 (6.5) | 0 (0) | 3 (6.7) | 3 (10) | 0.20 |

Data are presented as median [first through third quartiles] or number (%). AST = Aspartate Aminotransferase; CAP = Community-Acquired Pneumonia.

^a^ P values refer to differences between bacterial, viral, mixed and no etiology groups in univariate logistic regression.

**Table S3.** Multivariate analysis of the risk factors for hospital death in 174 patients with severe CAP.

| Variables | OR | 95% CI | P value |
| --- | --- | --- | --- |
| Microbiological diagnosis |  |  |  |
| Bacterial pneumonia | Ref | ... |  |
| Viral pneumonia | 0.80 | 0.22 - 2.93 | 0.74 |
| Mixed pneumonia | 1.96 | 0.61 - 6.31 | 0.26 |
| No etiology pneumonia | 1.69 | 0.38 - 7.60 | 0.49 |
| Transfer from another ward | 2.98 | 1.19 - 7.48 | 0.02 |
| Sodium > 140 mmol/L | 3.73 | 1.40 - 9.96 | 0.009 |
| PSI class IV-V at hospital referral | 19.06 | 2.43 - 149.4 | 0.005 |

OR = odds ratio; PSI = Pneumonia Severity Index; Ref = reference; 95% CI = 95% Confidence Interval. Transfer from another ward included transfers from another ICU and from the medical wards.

**Table S4.** Multivariate analysis of the risk factors for mechanical ventilation for more than 7 days in survivors at Day 28.

| Variables | OR | 95% CI | P value |
| --- | --- | --- | --- |
| Microbiological diagnosis |  |  |  |
| Bacterial pneumonia | Ref | ... |  |
| Viral pneumonia | 0.84 | 0.25 - 2.80 | 0.78 |
| Mixed pneumonia | 4.40 | 1.39 - 13.98 | 0.01 |
| No etiology pneumonia | 0.97 | 0.24 - 3.94 | 0.97 |
| Coronary artery disease | 4.19 | 1.22 - 14.34 | 0.02 |
| Shock on ICU admission | 5.86 | 1.94 - 17.68 | 0.002 |
| Alanine aminotransferase > 63 U/L | 2.95 | 1.08 - 8.02 | 0.03 |
| Glucose (mmol/L) | 1.12 | 1.01 - 1.23 | 0.03 |

OR = odds ratio; Ref = reference; 95% CI = 95% Confidence Interval.

**Table S5.** Baseline characteristics, behavior during ICU stay, and outcome of 45 patients with mixed infection, according to the viral diagnosis.

| Patients | Mixed  group  (n=45) | Mixed Non-Influenza subgroup  (n=30) | Mixed Influenza  subgroup  (n=15) | P  value ^a^ |
| --- | --- | --- | --- | --- |
| Age, y | 63 [54;75] | 59 [52 ; 74] | 68 [57 ; 78] | 0.33 |
| Chronic immunosuppression | 14 (31.1) | 12 (40) | 2 (13.3) | 0.07 |
| Chronic disease ^b^ | 20 (44.4) | 16 (53.3) | 4 (26.7) | 0.09 |
| Transfer from another ward ^c^ | 17 (37.8) | 12 (40) | 5 (33.3) | 0.66 |
| SAPS II score | 46 [34;59] | 46 [36 ; 61] | 43 [28 ; 55] | 0.53 |
| PSI score at hospital referral | 119 [98;126] | 111.5 [98 ; 124] | 125 [117 ; 141] | 0.12 |
| PSI class IV-V at hospital referral | 36 (80) | 24 (80) | 12 (80) | 1.00 |
| Organ supports during ICU stay |  |  |  |  |
| Non invasive ventilation | 12 (26.7) | 7 (23.3) | 5 (33.3) | 0.47 |
| Mechanical ventilation | 36 (80) | 24 (80) | 12 (80) | 1.00 |
| ARDS | 22 (48.9) | 15 (50) | 7 (46.7) | 0.83 |
| Dialysis | 12 (26.7) | 8 (26.7) | 4 (26.7) | 1.00 |
| Vasopressors | 27 (60) | 16 (53.3) | 11 (73.3) | 0.20 |
| Outcome |  |  |  |  |
| Length of mechanical ventilation, d | 9 [6;14] | 9.5 [5 ; 18.5] | 9 [7.5 ; 12.5] | 0.91 |
| Follow-up duration, d ^d^ | 16 [11;31] | 21 [10 ; 38] | 12 [11 ; 19] | 0.13 |
| Hospital mortality | 13 (28.9) | 7 (23.3) | 6 (40) | 0.24 |
| Complicated course ^e^ | 31 (68.9) | 19 (63.3) | 12 (80) | 0.25 |

Data are presented as median [first through third quartiles] or number (%).

ARDS= Acute Respiratory Distress Syndrome; ICU= Intensive Care Unit; PSI = Pneumonia Severity Index; SAPS = Simplified Acute Physiologic Score.

^a^ P values refer to differences between mixed non-influenza and mixed influenza subgroups in univariate logistic regression. ^b^ Chronic disease included chronic dialysis, mellitus diabetes requiring oral medication and /or insulin, chronic heart failure classified NYHA 3 or 4, cirrhosis, chronic respiratory failure requiring long term oxygen therapy and chronic immunosuppression. ^c^ Transfer from another ward included transfers from another ICU and from the medical wards. ^d^ The follow-up duration was defined as the time between the date of the mPCR and the date of hospital discharge. If the patient was deceased in hospital, the date of death was considered for hospital discharge. If the patient was not deceased in hospital and the date of hospital discharge was not available, the date of ICU discharge was considered for hospital discharge. ^e^ Complicated course was defined as hospital death and/or mechanical ventilation > 7 days.

**Table S6.** Baseline characteristics, initial biological findings and radiological patterns, ICU course and outcome in bacteria-matched patients with severe CAP.

| Patients | Bacterial  group  (n=33) | Mixed  group  (n=33) | P  value ^a^ |
| --- | --- | --- | --- |
| Age, y | 65 [53 ; 75] | 65 [52 ; 76] | 0.87 |
| Sex, male | 24 (72.7) | 24 (72.7) | 1.00 |
| Weight, kg | 69 [59 ; 76] | 70 [59 ; 77] | 0.92 |
| Smoking | 11 (36.7) | 9 (29) | 0.80 |
| Mc Cabe score > 1 | 4 (12.1) | 8 (24.2) | 0.34 |
| WHO Performans status > 0 | 7 (22.6) | 10 (34.5) | 0.18 |
| Chronic immunosuppression | 13 (39.4) | 10 (30.3) | 0.61 |
| HIV | 5 (15.2) | 3 (9.1) | 0.73 |
| Steroid therapy | 2 (6.1) | 3 (9.1) | 1.00 |
| Other immunosuppressive | 3 (9.1) | 5 (15.2) | 0.73 |
| Solid organ transplantation | 2 (6.1) | 3 (9.1) | 1.00 |
| Cancer | 5 (15.2) | 2 (6.1) | 0.45 |
| Chronic disease ^b^ | 15 (45.5) | 15 (45.5) | 1.00 |
| HCAP ^c^ | 16 (48.5) | 16 (48.5) | 1.00 |
| Transfer from another ward ^d^ | 8 (24.2) | 11 (33.3) | 0.58 |
| Antibiotics before ICU admission ^e^ | 8 (24.2) | 9 (27.3) | 1.00 |
| Organ failures on ICU admission |  |  |  |
| Glasgow < 15 | 7 (21.2) | 11 (33.3) | 0.39 |
| Shock | 11 (33.3) | 9 (27.3) | 0.80 |
| PaO_2_/FIO_2_ ratio | 178 [139;230] | 158 [131; 233] | 0.90 |
| SAPS II score | 38 [32;56] | 46 [36;61] | 0.38 |
| PSI score at hospital referral | 109 [84;155] | 118 [98;125] | 0.70 |
| PSI class IV-V at hospital referral | 21 (63.6) | 27 (81.8) | 0.11 |
| Initial biological findings |  |  |  |
| Neutrophils, G/L | 11.2 [7.3 ; 18.5] | 8 [3.3 ; 11.7] | 0.06 |
| Lymphocytes, G/L | 0.9 [0.6 ; 1.4] | 0.7 [0.4 ; 1.2] | 0.30 |
| Platelets, G/L | 210 [163 ; 262] | 187 [133 ; 253] | 0.20 |
| Procalcitonin, µg/L | 4.6 [0.7 ; 26.5] | 14 [0.4 ; 74.4] | 0.26 |
| Creatine kinase, U/L | 113 [72.5 ; 222] | 411 [188 ; 898] | <0.01 |
| Lactate dehydrogenase, U/L | 278 [182 ; 447] | 492 [353 ; 723] | 0.06 |
| AST, U/L | 41 [26 ; 55] | 40 [34 ; 119] | 0.42 |
| Bilirubin total, µmol/L | 12 [8 ; 19] | 13 [9 ; 18] | 0.74 |
| Cardiac troponin T, > 0.35 ng/mL | 3 (10.3) | 3 (11.1) | 1.00 |
| Initial radiologic patterns |  |  |  |
| Bilateral infiltrate | 22 (66.7) | 21 (63.6) | 1.00 |
| Alveolar-interstitial infiltrates | 9 (27.3) | 18 (60) | <0.01 |
| Pleural effusion | 3 (9.1) | 2 (6.1) | 1.00 |
| Organ supports during ICU stay |  |  |  |
| Non invasive ventilation | 10 (30.3) | 7 (21.2) | 0.55 |
| Mechanical ventilation | 19 (57.6) | 26 (78.8) | 0.14 |
| ARDS | 12 (36.4) | 17 (51.5) | 0.30 |
| Dialysis | 7 (21.2) | 8 (24.2) | 1.00 |
| Vasopressors | 16 (48.5) | 21 (63.6) | 0.30 |
| Outcome |  |  |  |
| Duration of mechanical ventilation, d | 5 [3 ; 17] | 9.5 [6 ; 15] | 0.72 |
| Follow-up duration, d ^f^ | 14 [5 ; 19] | 15 [11 ; 31] | 0.15 |
| Hospital mortality | 4 (12.1) | 11 (33.3) | 0.07 |
| Complicated course ^g^ | 12 (36.4) | 23 (69.7) | <0.01 |

Data are presented as median [first through third quartiles] or number (%).AST = Aspartate Aminotransferase; ARDS = Acute Respiratory Distress Syndrome; CAP = Community-Acquired Pneumonia; HCAP = Health –Care Associated Pneumonia; HIV = Human Immunodeficiency Virus; ICU= Intensive Care Unit; PSI = Pneumonia Severity Index; SAPS = Simplified Acute Physiologic Score; WHO = World Health Organization.

^a^ P values refer to differences between bacterial and mixed groups in univariate logistic regression. ^b^ Chronic disease included chronic dialysis, mellitus diabetes requiring oral medication and /or insulin, chronic heart failure classified NYHA 3 or 4, cirrhosis, chronic respiratory failure requiring long term oxygen therapy and chronic immunosuppression. ^c^ Pneumonia was considered health-care associated (HCAP) in the presence of at least one of the following conditions: hospitalization for ≥ 2 days in the preceding 90 days, institutionalization, chronic dialysis and chronic immunosuppression. ^d^ Transfer from another ward included transfers from another ICU and from the medical wards. ^e^ Antibiotics before ICU admission referred to any administration of antibiotics, whatever drug regimen, before the ICU referral, i.e in emergency departments or in other medical wards. ^f^ The follow-up duration was defined as the time between the date of the mPCR and the date of hospital discharge. If the patient was deceased in hospital, the date of death was considered for hospital discharge. If the patient was not deceased in hospital and the date of hospital discharge was not available, the date of ICU discharge was considered for hospital discharge. ^g^ Complicated course was defined as hospital death and/or mechanical ventilation > 7 days.

**Subgroup analysis of bacteria-matched patients** Thirty three bacteria-matched pairs have been formed. *S. pneumoniae* was the predominant bacterium (18/33, 54.5%). Except one patient, controls were coinfected with only one viral specie, with predominance of picornavirus (11/33, 33.3%) and influenza viruses (9/33, 27.3%). In univariate logistic regression, the matched analysis showed a trend toward a higher severity in mixed group, illustrated by a higher frequency of PSI class IV-V at hospital referral (81.8% vs 63.6%, p=0.11) (**Table S3**). Virus-bacteria coinfected patients exhibited higher serum creatine kinase (411 vs 113 U/L, p<0.01) and higher frequency of alveolar-interstitial infiltrates (60% vs 27%, p<0.01). No statistical difference were seen in terms of organ failures and death, despite a trend toward an increased incidence of mechanical ventilation (78.8% vs 57.6%, p=0.14) and a higher hospital mortality (33.3% vs 12.1%, p=0.07) in mixed group. Finally, a complicated course was observed in 23 (69.7%) patients of the mixed group, as compared to 12 (36.4%) patients of the bacterial group (p<0.01). In multivariate analysis, the viral-bacterial coinfection was shown as independently associated with the complicated course (OR, 5,907; CI 95%, 1.288 – 27.083; p=0.02). Conversely, the viral-bacterial coinfection was not shown independently associated with the hospital death (OR, 4.64; CI 95%, 0.88 – 24.5; p=0.07).

An additional analysis was performed with limiting the mixed group to the non-influenza infected-patients (24 bacteria-matched pairs). In multivariate analysis, the viral-bacterial coinfection was still shown as independently associated with the complicated course (OR, 9.688; CI 95%, 1.174– 79.925; p=0.03).
